# Anti-PD-1 increases the clonality and activity of tumor infiltrating antigen specific T cells induced by a potent immune therapy consisting of vaccine and metronomic cyclophosphamide

**DOI:** 10.1186/s40425-016-0169-2

**Published:** 2016-10-18

**Authors:** Genevieve M. Weir, Olga Hrytsenko, Tara Quinton, Neil L. Berinstein, Marianne M. Stanford, Marc Mansour

**Affiliations:** 1 Immunovaccine Inc., 1344 Summer St., Halifax, NS B3H 0A8 Canada; 2 Sunnybrook Research Institute, 2075 Bayview Ave., Toronto, ON M4N 3M5 Canada; 3 University of Toronto, 27 King’s College Cir, Toronto, ON M5S 1A1 Canada; 4 Department of Microbiology & Immunology, Dalhousie University, 5850 College St., Room 7C, Halifax, NS B3H 4R2 Canada

**Keywords:** Vaccine, Metronomic cyclophosphamide, PD-1, Tumor microenvironment, Clonality

## Abstract

**Background:**

Future cancer immunotherapies will combine multiple treatments to generate functional immune responses to cancer antigens through synergistic, multi-modal mechanisms. In this study we explored the combination of three distinct immunotherapies: a class I restricted peptide-based cancer vaccine, metronomic cyclophosphamide (mCPA) and anti-PD-1 treatment in a murine tumor model expressing HPV16 E7 (C3).

**Methods:**

Mice were implanted with C3 tumors subcutaneously. Tumor bearing mice were treated with mCPA (20 mg/kg/day PO) for seven continuous days on alternating weeks, vaccinated with HPV16 E7_49-57_ peptide antigen formulated in the DepoVax (DPX) adjuvanting platform every second week, and administered anti-PD-1 (200 μg/dose IP) after each vaccination. Efficacy was measured by following tumor growth and survival. Immunogenicity was measured by IFN-γ ELISpot of spleen, vaccine draining lymph nodes and tumor draining lymph nodes. Tumor infiltration was measured by flow cytometry for CD8α^+^ peptide-specific T cells and RT-qPCR for cytotoxic proteins. The clonality of tumor infiltrating T cells was measured by TCRβ sequencing using genomic DNA.

**Results:**

Untreated C3 tumors had low expression of PD-L1 in vivo and anti-PD-1 therapy alone provided no protection from tumor growth. Treatment with DPX/mCPA could delay tumor growth, and tri-therapy with DPX/mCPA/anti-PD-1 provided long-term control of tumors. We found that treatment with DPX/mCPA/anti-PD-1 enhanced systemic antigen-specific immune responses detected in the spleen as determined by IFN-γ ELISpot compared to those in the DPX/mCPA group, but immune responses in tumor-draining lymph nodes were not increased. Although no increases in antigen-specific CD8α^+^ TILs could be detected, there was a trend for increased expression of cytotoxic genes within the tumor microenvironment as well as an increase in clonality in mice treated with DPX/mCPA/anti-PD-1 compared to those with anti-PD-1 alone or DPX/mCPA. Using a library of antigen-specific CD8α^+^ T cell clones, we found that antigen-specific clones were more frequently expanded in the DPX/mCPA/anti-PD-1 treated group.

**Conclusions:**

These results demonstrate how the efficacy of anti-PD-1 may be improved by combination with a potent and targeted T cell activating immune therapy.

**Electronic supplementary material:**

The online version of this article (doi:10.1186/s40425-016-0169-2) contains supplementary material, which is available to authorized users.

## Background

Immune based cancer treatments are beginning to show promise in clinical trials and have already begun to integrate into the standard of care for multiple indications, with many others sure to follow. Different types of immune therapies have complimentary mechanisms of action, and when combined rationally could be used to overcome complex immune suppressive networks utilized by tumors [1]. Cancer vaccines generate active and targeted immune responses, and could be a cornerstone of these combinations [2, 3].

DepoVax™ (DPX) is a water free lipid-in-oil vaccine formulation with demonstrated ability to enhance responses to peptide vaccines in animals and in human clinical trials [4–6]. The DPX formulation utilizes lipids to effectively combine multiple peptide or protein antigens and adjuvants into an oil phase, resulting in a formulation that uniquely provides a long lasting and immunogenic depot in vivo [7]. Peptides formulated in DPX generate a stronger antigen-specific immunity compared to other types of emulsion-based depot forming vaccine platforms (unpublished data). Previously, we evaluated metronomic cyclophosphamide (mCPA) as an immune modulator with DPX vaccination in a preclinical tumor model [6]. We found that the treatment combination provided enhanced therapeutic control of tumors, which could be attributed to an enrichment in the expansion of vaccine-induced antigen specific CD8^+^ T cells. These results were translated to a Phase 1/1b clinical trial evaluating DPX-Survivac, a DPX vaccine containing multiple peptide antigens derived from the tumor associated protein survivin (DPX-Survivac), and mCPA in advanced ovarian cancer patients [5]. The immune responses induced by patients treated with DPX-Survivac and mCPA were significantly higher than responses induced by patients treated with DPX vaccine alone. Although CD4^+^FoxP3^+^ regulatory T cell (Treg) reduction has been a reported effect of administering low dose cyclophosphamide, we and others have not detected any reduction in the Treg population following administration [5, 8, 9]. Treg depletion is one of many immune modulating effects attributed to low dose cyclophosphamide [10], and the effects of the treatment may be specific to the type of cancer involved. Due of the multiple effects of cyclophosphamide on the immune system, combinations with other forms of immune therapy must be individually assessed.

Immune therapies are likely to benefit by combining multiple agents that target complimentary mechanisms of the immune system [1, 3]. Although we have demonstrated that the DPX/mCPA combination is effective in inducing antigen-specific CD8^+^ T cells that could effectively control small tumors in our preclinical model, the efficacy was limited in the treatment of more advanced tumors [6]. This may be due to increased immune suppression exerted by advanced tumors, and may also be a barrier for clinical applications. Therefore, in this work we have explored additional immune therapies that may enhance the immune response induced by DPX/mCPA through complimentary mechanisms in order to identify promising combinations for clinical testing and novel immune biomarkers. In our preclinical work, programmed death-1 (PD-1) and programmed death ligand-1 (PD-L1) were both increased within the tumor by DPX/mCPA treatment, corresponding to increases in cytotoxic genes such as IFN- γ and granzyme B. PD-1 is a co-inhibitory receptor expressed primarily by activated lymphocytes which induces tolerance/exhaustion upon interaction with its ligand PD-L1. The expression of PD-1 on T cells is increased in response to persistent antigen exposure [11]. PD-L1 is frequently upregulated in many tumor types, particularly in response to IFN-γ, and is a mechanism of immune suppression used by tumors to escape immune detection [12, 13]. The biological significance of this mechanism was demonstrated through the substantial clinical benefit observed in Phase 3 testing of the anti-PD-1 monoclonal antibodies nivolumab and pembrolizumab in patients with advanced cancers, where anti-tumor responses to these antibodies correlated with the presence of tumor infiltrating lymphocyte (TIL) activity [14, 15]. Encouraged by these results, other monoclonal antibodies and small molecule drugs that block PD-1 signaling are in various stages of development [16].

Increased PD-1 expression has been correlated with high mutation load tumors which bear immunogenic neoantigens [17, 18]. Indeed, within the tumor microenvironment (TME) of clinical samples, PD-1 expression is primarily induced on tumor-specific T cells [19, 20]. These observations suggest that anti-PD-1 therapy would be most effective in patients that have spontaneous tumor-specific immune responses with increased numbers of TILs, presumably to immunogenic neoantigens [21]. However, this mutation load and these immune responses do not exist in all patients, nor in all cancer types, to the same extent, therefore anti-PD-1 therapy is not uniformly effective. Using preclinical models, others have shown that combining anti-PD-1 therapy with vaccination has a synergistic effect in increasing tumor infiltrating immune cells in non-PD-1/PD-L1 expressing tumors, resulting in better efficacy [22–24]. It has been postulated that this synergistic effect may be in part due to expansion of T cell clones specific to other tumor antigens besides those in the vaccine, the “epitope spreading” phenomenon [22, 23]. However, this has not been directly evaluated.

In this study, we evaluate the combination of three distinct immune therapies, DPX vaccination, mCPA and anti-PD-1, in the HPV16 E7 expressing C3 preclinical tumor model. The advantage to using this model is that there is an immunodominant CD8^+^ T cell epitope, HPV16E7_49-57_ (R9F), and R9F-specific CD8^+^ T cells can be induced by vaccination. This allowed us to evaluate the antigen-specific CD8^+^ T cell responses and clonality using a defined target. Our results provide a mechanistic rationale for the combination of these therapies to improve treatment of advanced tumors, and also demonstrate how clonality may be used to assess the quality of antigen-specific immune responses.

## Methods

### Mice and tumor implantation

Pathogen-free, 6–8 week old female C57BL/6 mice were obtained from Charles River Labs (St. Constant, PQ, Canada). Mice were housed under filter-top conditions and provided food and water ad libitum.

The C3 cell line, provided by Dr. Martin Kast (USC, Los Angeles, USA), is derived from C57BL/6 mouse embryo cells transfected to express HPV16 [25]. The C3 tumor line was maintained in IMDM (Gibco) supplemented with 10 % fetal bovine serum (FBS; HyClone) 2 % penicillin-streptomycin (Gibco), 50 mM mercaptoethanol (Gibco) and 2 mM L-glutamine (Gibco). Mice were implanted with 3 × 10^5^ C3 tumor cells subcutaneously in the left flank.

Tumor growth was measured with digital calipers twice weekly and tumor volume calculated using the formula [(*width* ^2^ × *length*)/2]. For experiments requiring determinations of survival, endpoint was determined to be when mice had tumor volumes of ≥2000 mm^3^, or showed significant signs of ill health, such as wasting, severe dehydration, significant decrease in activity and hunched or prostate posture. When endpoint was determined, mice were humanely euthanized per CCAC guidelines.

### Peptides

All peptides were synthesized by NeoMPS (San Diego, CA, USA) at >90 % purity. The H2Db peptide epitope HPV16E7_49-57_ (RAHYNIVTF; R9F) was used in each study. In some studies, the irrelevant H2Db peptide epitope WT-1_126-134_ (RMFPNAPYL; R9L) was used. All vaccines contained a universal T helper peptide PADRE (AKXVAAWTLKAA).

### Vaccine preparation and immunization

Peptides were formulated in DepoVax vaccines with a proprietary adjuvant as previously described [26]. Briefly, peptides and adjuvant were solubilized in appropriate buffer and mixed with 10:1 (w:w) DOPC/cholesterol mixture (Lipoid GmBH, Germany) to form liposomes. The aqueous mixture was lyophilized to a dry cake which was reconstituted with Montanide ISA51 VG (SEPPIC, France) just prior to injection. Mice were vaccinated subcutaneously on the right flank with 50 μl of vaccine. Each dose of vaccine contained 10 μg R9F fused to PADRE + 20 μg adjuvant. When multiple vaccinations were administered they were given in the same area but avoiding previous immunization sites.

### Cyclophosphamide treatment

Cyclophosphamide (Sigma-Aldrich) was reconstituted in PBS and provided for seven consecutive days in drinking water (PO) at 0.133 mg/mL, calculated to deliver 20 mg/kg/day based on 3 mL water/mouse/day. Water was changed every 2–3 days. Mice that were treated with cyclophosphamide were monitored daily for signs of ill health indicating adverse reactions to cyclophosphamide treatment.

### Antibody treatment

Monoclonal antibodies for in vivo administration were purchased from BioXCell (West Lebanon, NH, USA). Anti-PD-1 (clone RMP1-14) or isotype control (clone 2A3) was administered as a 200 μg dose by intraperitoneal injection on days indicted.

### IFN-γ ELISpot

IFN-γ ELISpot was performed as described previously [6]. Briefly, mature dendritic cells (DCs) were generated by culturing bone marrow cells from naïve C57BL/6 mice in complete RPMI media [RPMI 1640 (Gibco) + 10 % FBS, 2 % penicillin/streptomycin (Gibco), 2 mM L-glutamine (Gibco), 50 mM β-mercaptoethanol (Sigma-Aldrich), and 5 mM HEPEs buffer (Gibco)] supplemented with murine GM-CSF (Peprotech). DCs were loaded with 20 μg/mL peptides on day 7. Day 8 DCs were used as antigen presenting cells for ELISPOT and were resuspended in complete RPMI at 2 × 10^5^ cells/mL.

Right (vaccine draining) and left (tumor draining) inguinal lymph nodes were collected from mice upon termination. Single cell suspensions were prepared in complete RPMI media and cell concentration adjusted to 2 × 10^6^ cells/mL. Lymph node cells (100 μL) and DCs (100 μL) were added to IFN-γ ELISpot plates (BD Bioscience). The ELISpot plate was incubated overnight at 37 °C, 5 % CO_2_ and then developed the next day using AEC kit (Sigma-Aldrich). Spots were counted using ELISpot Reader (C.T.L. Ltd, Shaker Heights, OH, USA) and enumerated as number of spot-forming units (SFU) per well.

IFN-γ ELISpot performed using splenocytes had the following modifications. Single cell suspensions of splenocytes were prepared by lysing RBCs with ammonium-chloride-potassium solution and resuspending cells at 5 × 10^6^ cells/ mL in complete RPMI media. A volume of 100 μL cells was added into IFN-γ ELISpot plate and stimulated with 100 μL complete RPMI containing no peptide (background control), 20 μg/mL R9F or irrelevant peptide, or 5 × 10^5^ cells/mL C3 tumor cells.

### Tumor dissociation

Tumors were extracted from mice upon termination and chopped into small pieces using a scalpel. Pieces were transferred into a 15 mL tube containing 5–10 mL of digestion buffer [1 mg/mL collagenase type 1 (Gibco) + 0.1 mg/mL DNase I (Sigma) in RPMI 1640] and incubated in a shaker at 37 °C for 30 min. Suspensions were then strained into a new tube through 40 μM filter. Cells were washed in PBS and used for flow cytometry.

### Flow cytometry and FACS

Cells were pre-incubated with normal rat serum to block non-specific staining. Antibody cocktails were added and cells incubated at 4 °C for 30 min. The following fluorochrome conjugated anti-mouse antibodies were used CD3 (145-2C11), CD4 (GK1.5), CD8α (53.6-7), CD45 (30 F11), all purchased from eBioscience. R9F-dextramer-PE was obtained from Immudex. A FASCcalibur (BD Bioscience) was used for acquisition of flow cytometry data and analysis was performed using WinList 7.0 (Verity Software, Topsham, ME, USA). FACS sorting was performed using a FACSAria III (BD Bioscience) and FACSDiva 6.0 software.

### RT-qPCR

Total RNA was isolated using RNeasy Mini Kit (QIAGEN); 4 μg aliquots were treated with DNAse I (Invitrogen) and reverse transcribed using a SuperScript III reverse transcriptase kit (Invitrogen) and oligo(dT) primer (Invitrogen). PCR primers for *Cd8a*, *Gzmb*, *Ifng*, *Prf*, *Tbx21*, *CD4*, *Pdcd1*, *CD274*, *GATA3* and *Gzmb* were designed using Primer-BLAST algorithm (Additional file 1: Table S1). Amplifications of these transcripts were performed on a Rotor-Gene Q real-time PCR machine using a QuantiFast SYBR Green PCR kit (QIAGEN). Data were analyzed based on the standard curve method and normalized against levels of GAPDH mRNA.

### TCRβ sequencing

Tumor genomic DNA was extracted using the DNeasy Blood and Tissue Kit (Qiagen). CD8α^+^ R9F-specific T cells were purified by FACS using R9F-dextramer reagent, anti-CD8α and anti-CD3. The cells were pelleted, frozen at -80 °C and sent to Adaptive Biotechnologies. The TCRβ locus was sequenced using the ImmunoSEQ survey level assay by Adaptive Biotechnologies (Seattle, WA). TCRβ sequencing was analyzed using the ImmunoSEQ Analyzer (Adaptive Biotechnologies).

### Statistical analysis

Statistical analysis was conducted with GraphPad Prism 6 (La Jolla, CA, USA) software. Data was analysed by appropriate tests as indicated in figure legends. Significance denoted as: *p ≤ 0.05, **p ≤ 0.01, ***p ≤ 0.001, ****p ≤ 0.0001.

## Results

### PD-1 and PD-L1 expression is induced in C3 tumors in response to infiltration with tumor-specific CD8^+^ T cells

**Fig. 1:**
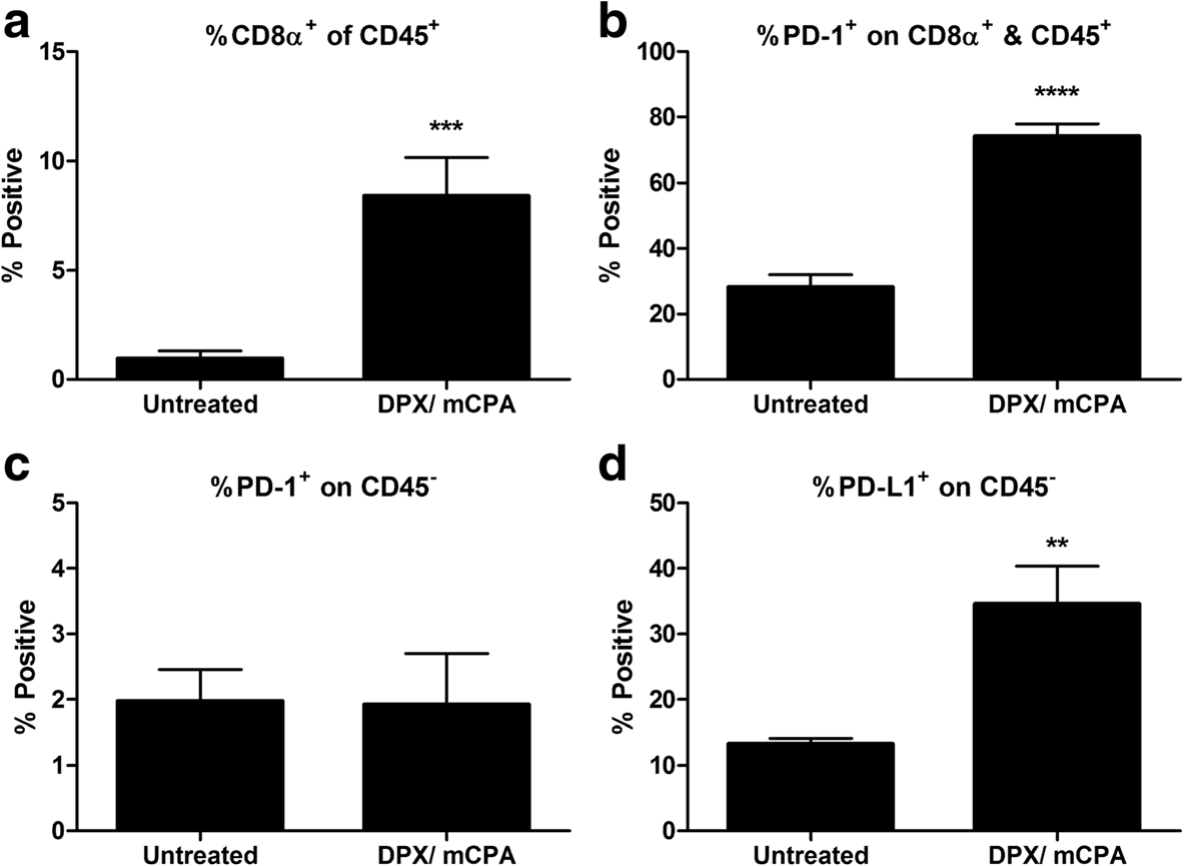
DPX vaccination and mCPA treatment increased infiltration of C3 tumors with PD-1^+^ CD8α^+^ T cells. Mice bearing C3 tumors were treated with mCPA and vaccinated with DPX-R9F. Eight days after vaccination, mice were terminated. Tumors were dissociated and analysed by flow cytometry for expression of CD45, CD8a, PD-1 and PD-L1. **a** Percent CD8α positive of CD45 positive cells; **b** Percent PD-1 positive of CD8α&CD45 double positive cells; **c** Percent PD-1 positive of CD45 negative cells; **d** Percent PD-L1 positive of CD45 negative cells Results pooled from two separate experiments, *n* = 6–8, average ± SEM, statistics by students t-test, ** *p* < 0.01, ***p < 0.0001

In our previous work, we detected enhanced expression of PD-1 mRNA in HPV16 E7 expressing C3 tumors after treatment with a DPX vaccine containing HPV16E7_49-57_ peptide (DPX-R9F) and mCPA [6]. To evaluate if the increased PD-1 expression in the tumors was due to increased accumulation of CD8^+^ T cells, we performed a flow cytometry analysis on TILs. Mice were implanted with C3 tumors and were treated with mCPA for seven consecutive days starting 2 weeks after implantation, at the time when most tumors become palpable. At the end of the week, mice were vaccinated once with DPX-R9F, and all mice were euthanized 8 days later. The dissociated tumor preparations from treated mice had significantly increased infiltration by CD45^+^CD8^+^ T cells compared to untreated mice (Fig. 1a). PD-1 expression on the CD45^+^CD8^+^ T cells from these treated mice was also significantly elevated (Fig. 1b), and yet PD-1 expression on non-leukocytes (i.e. CD45 negative) was unchanged (Fig. 1c). Correspondingly, PD-L1 expression on non-leukocytes was increased in treated mice (Fig. 1d). Additionally, we found that C3 cells treated in vitro with IFN-γ increased expression of PD-L1, but not PD-1 or PD-L2 (Additional file 1: Figure S1).

### PD-1 blockade enhanced the efficacy of DPX vaccination with mCPA

**Fig. 2:**
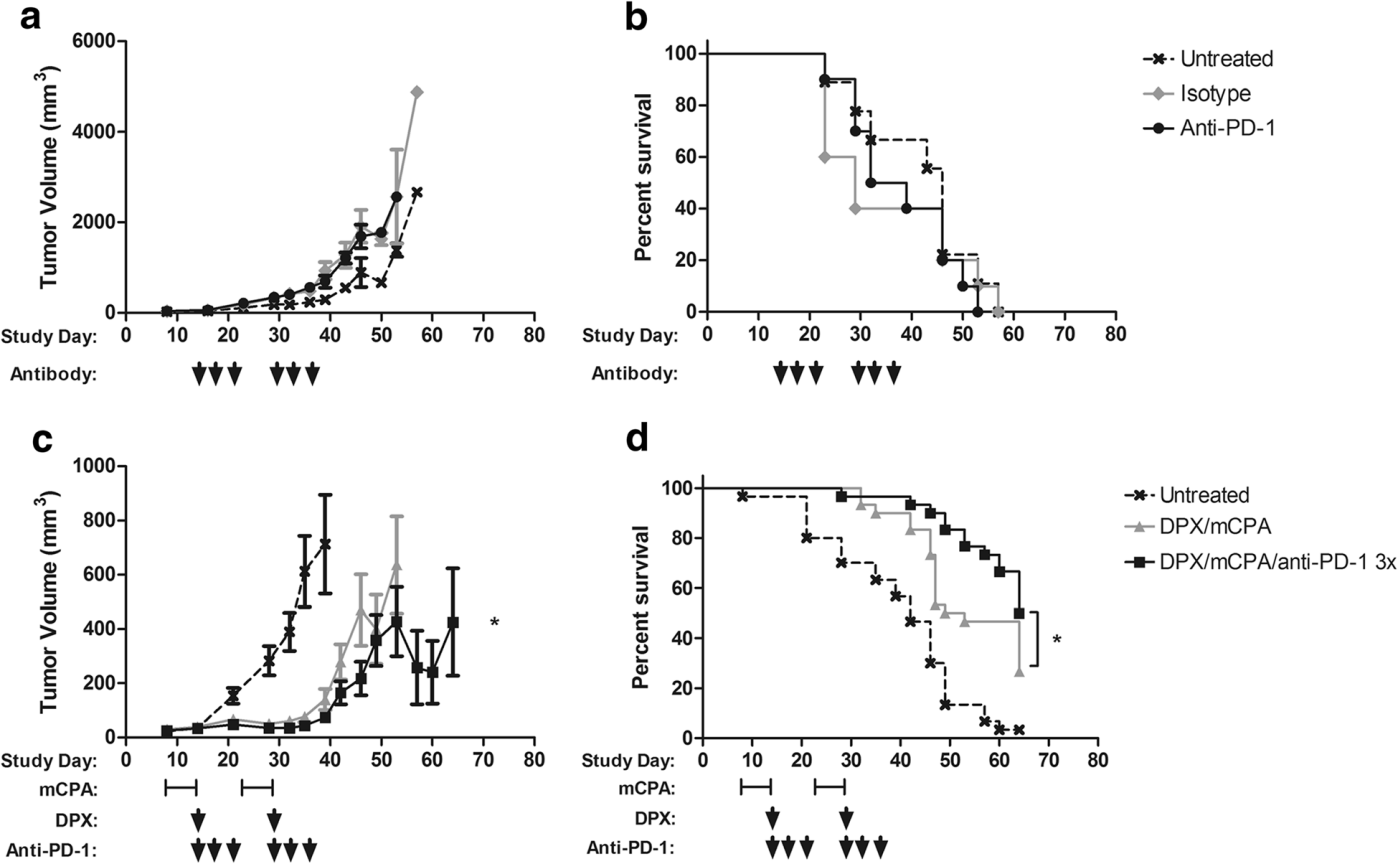
Anti-PD-1 treatment is ineffective alone, but increases efficacy of DPX vaccination and mCPA. Mice were implanted with C3 tumors and treated with isotype control antibody (*n* = 10) or anti-PD-1 (*n* = 10) on study days 15, 18, 21, 29, 32, 35; average tumor volume **a** and survival **b**. Mice bearing C3 tumors were treated with mCPA for 1 week starting 8 and 22 days after implantation and vaccinated with DPX-R9F on days 15 and 29 (*n* = 30), anti-PD-1 was provided on study days 15, 18, 21, 29, 32, 35 (*n* = 30); average tumor volume **c** and survival **d**. Statistics of tumor volume by linear regression and survival by Mantel-Cox, *p < 0.05

To determine if the C3 model was responsive to anti-PD-1 therapy, C57BL6 mice (*n* = 10) were implanted with C3 tumors. Anti-PD-1 therapy, or isotype control, was initiated 15 days later when tumors were established, and administered three times on days 15, 18, and 21. Treatment was repeated 2 weeks later. In our experience with this model, initiating treatment on these days provides suboptimal protection from tumor growth therefore we considered this to represent more advanced tumors. As shown in Fig. 2a and b, isotype control or anti-PD-1 therapy provided no delay from tumor growth or enhanced survival of mice. In a separate experiment, we also evaluated the combination of mCPA and anti-PD-1 without vaccination, however this combination did not provide any significant protection from tumor in this model (data not shown).

To assess if PD-1 blockade using anti-PD-1 monoclonal antibodies could enhance the efficacy of immunotherapy provided by DPX-R9F vaccination and mCPA, we tested the triple combination therapy in the C3 tumor model in three separate experiments each with 9–10 mice per group. The pooled results are shown in Fig. 2c and d. Mice implanted with C3 tumors were treated with mCPA starting 8 days after implantation. After 1 week of treatment, on study day 15, mice were vaccinated with DPX-R9F. Anti-PD-1 was administered on study days 15, 18 and 21. This treatment was repeated again 2 weeks later, starting on study day 22. Anti-PD-1 in combination with DPX-R9F/mCPA immunotherapy resulted in a significant delay in tumor growth and a significant increase in mouse survival compared to mice treated with DPX-R9F/mCPA. Similar results were obtained using two other tumor models, B16-F10 and HLA-A2^+^ ovarian tumors (Additional file 1: Figure S2).

### Anti-PD-1 increased the systemic immune response to DPX vaccination with mCPA

**Fig. 3:**
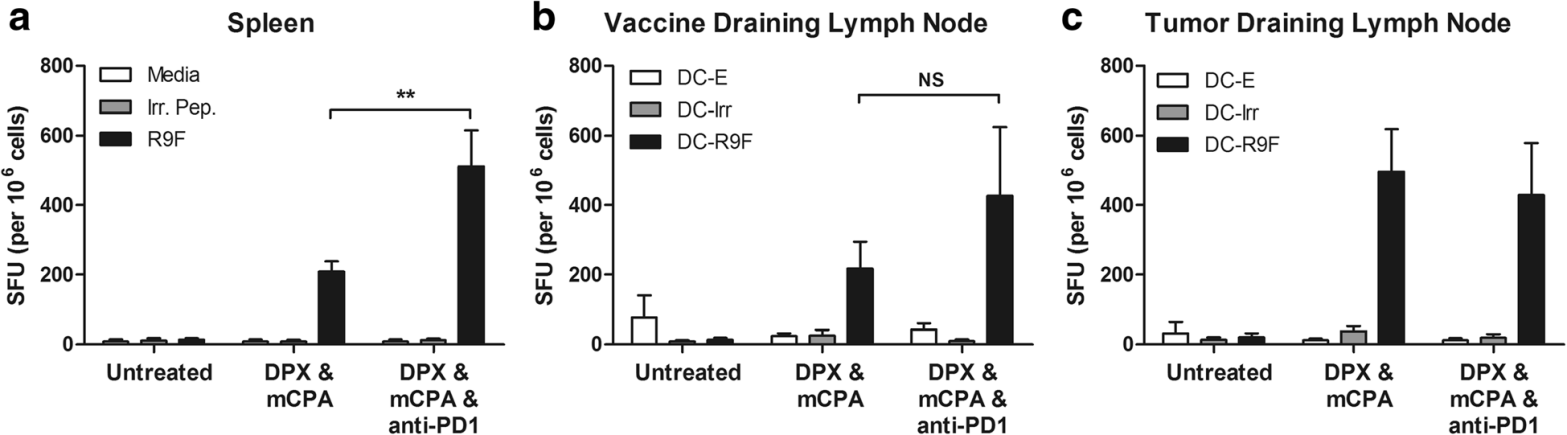
IFN-γ ELISpot analysis of C3 tumor bearing mice treated with vaccination, metronomic cyclophosphamide, and anti-PD-1. **a** Responses in the spleen **b** Responses in the vaccine draining lymph node **c** responses in the tumor draining lymph node. DC-E: Unloaded (empty) dendritic cells; DC-Irr: dendritic cells loaded with irrelevant (R9L) peptide; DC-R9F: dendritic cells loaded with R9F peptide. Results representative of at least two separate experiments, *n* = 3–5, statistics by 2-way ANOVA with Tukey post-test, **p < 0.01

To determine if anti-PD-1 enhanced the efficacy of treatment by increasing the immunogenicity of the vaccine, we performed an IFN-γ ELISpot assay using C3 tumor bearing mice. To ensure that mice would have tumors at the time of evaluation, we started treatment with mCPA 2 weeks after implantation and provided a single vaccination on day 21 and anti-PD-1 treatment on day 27. Mice were euthanized on study day 31, which was 10 days after vaccination. In order to characterize the vaccine-induced immune response, splenocytes, vaccine draining inguinal lymph node cells and tumor draining inguinal lymph node cells were assessed separately. The immune responses detected in the spleen were significantly higher when mice were treated with DPX/mCPA/anti-PD-1 compared to when mice were treated with DPX/mCPA (Fig. 3a). In the vaccine draining lymph nodes, a trend of higher immune responses in the DPX/ mCPA/anti-PD-1 treatment group was observed, but this increase was not significant (Fig. 3b). In the tumor draining lymph nodes the immune responses were the same in the two vaccinated groups (Fig. 3c), suggesting that a similar occupancy of vaccine induced CD8^+^ T cells was achieved in the tumor draining lymph nodes despite having a higher systemic immune response in the triple combination treatment group.

### Anti-PD-1 did not enhance accumulation of TILs induced by DPX vaccination and mCPA

**Fig. 4:**
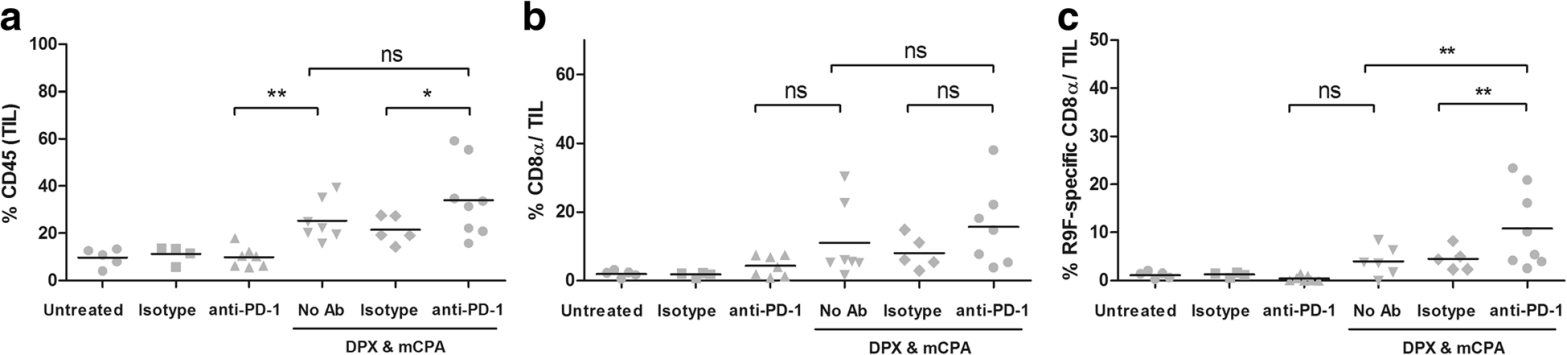
Anti-PD-1 increases tumor infiltration with antigen-specific CD8α^+^ T cells. Mice were implanted with C3 tumors and treated with 1 week of mCPA commencing 14 days after implantation. Mice were vaccinated on study day 21 and treated with anti-PD-1 or isotype control on study day 26. All mice were terminated on study day 31. **a** Percent tumor infiltrating leukocytes (TIL) defined as % CD45 positive cells; **b** Percent CD8α positive T cells in the TIL population; **c** Percent R9F-specific CD8α T cells in the TIL population. Results pooled from two separate experiments, *n* = 4–8, statistics by 1-way ANOVA with LSD post-test, *p < 0.05, **p < 0.01; ns: not significant (*p* > 0.05)

As the tumor draining lymph nodes did not exhibit the same increase in antigen-specific IFN-γ response as detected in the spleen, we postulated that perhaps they were being routed into the tumor where they could accumulate. We thus evaluated the TIL population using flow cytometry of dissociated tumor cells. We found that anti-PD-1 treatment alone did not increase the infiltration of C3 tumors with CD45^+^ TILs over those of untreated or isotype control treated mice (Fig. 4a). The combination of DPX-R9F and mCPA treatment resulted in a significant increase in CD45^+^ cells (25.3 %) as compared to that of anti-PD-1 treatment alone (9.8 %). Infiltration higher with DPX-R9F, mCPA and anti-PD-1 (34.1 %), but was not significant compared to DPX-R9F/ mCPA (Fig. 4a). A similar trend was observed for CD8α^+^ T cells (Fig. 4b). These results were consistent with expression patterns detected by immunohistochemistry of tumor sections using anti-CD45 and anti-CD8α (Additional file 1: Figure S2).

R9F antigen-specific CD8α^+^ T cells were detected using a multimer reagent. As shown in Fig. 4c, the numbers of R9F-specific CD8α^+^ T cells in the untreated, isotype treated and anti-PD-1 treated groups were consistently low (<1.5 % of CD45^+^ cells). The number of antigen-specific CD8α^+^ T cells was higher in the DPX-R9F/mCPA treated group (4.0 %) compared to that in the anti-PD-1 (0.5 %). The highest accumulation of R9F-specific CD8α^+^ T cells was detected in the group treated with the DPX-R9F/mCPA/anti-PD-1 combination (10.8 %), a significant increase compared to that in the DPX-R9F/mCPA treated group.

### Anti-PD-1 did not significantly increase the activity of tumor infiltrating antigen-specific CD8α^+^ T cells induced by DPX vaccination and mCPA

**Fig. 5:**
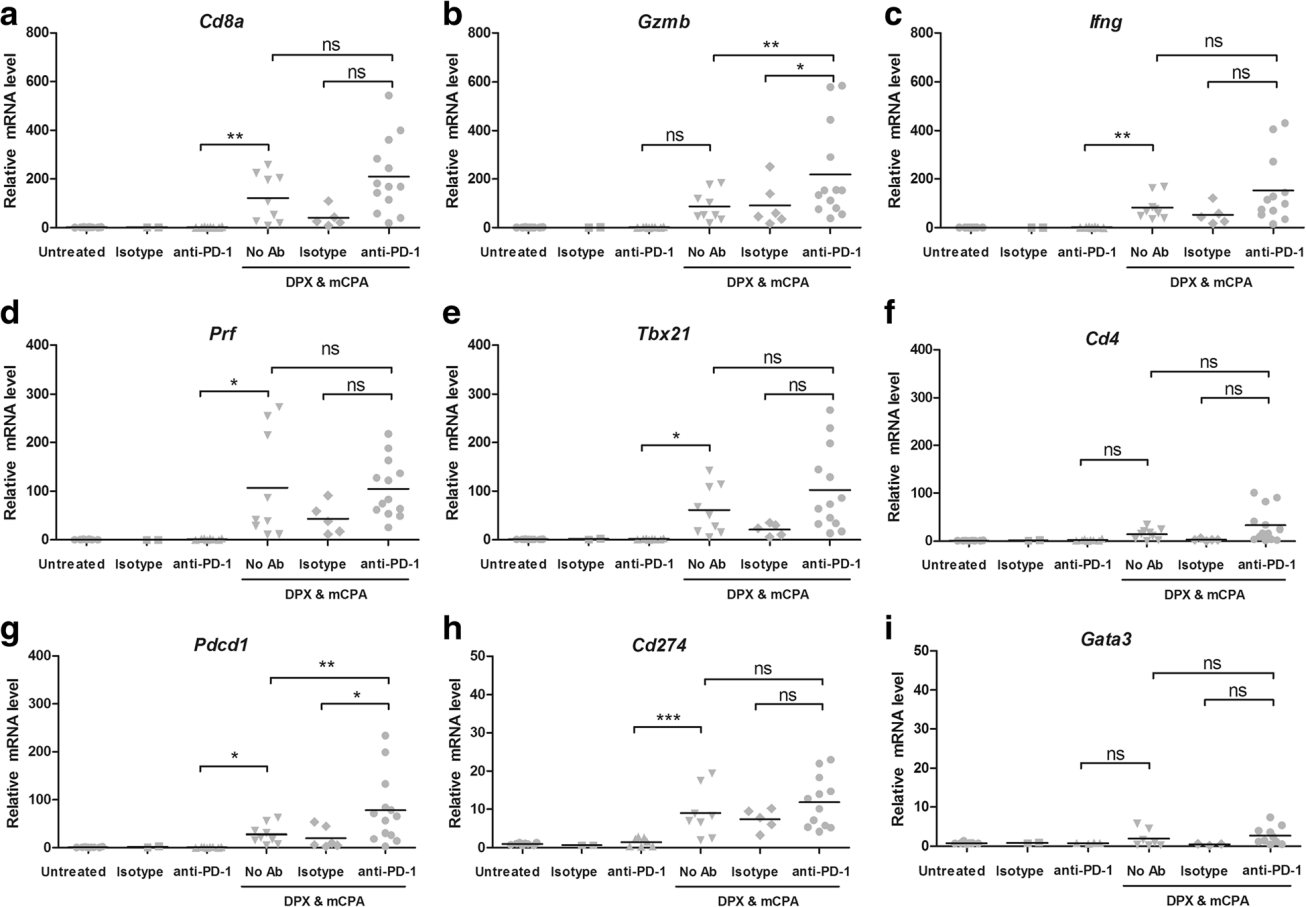
Expression of cytotoxic genes in tumour tissue after treatment with DPX vaccination, mCPA and anti-PD-1 by RT-qPCR. Mice were implanted with C3 tumors and treated with 1 week of mCPA commencing 14 days after implantation. Mice were vaccinated on study day 21 and treated with anti-PD-1 or isotype control on study day 26. All mice were terminated on study day 31. Total tumor mRNA analysed for gene expression by RT-qPCR, results normalized to the level of GAPDH mRNA and presented as fold of increase in mRNA level over the untreated control that was arbitrary set as 1. **a** *Cd8a* (CD8α), **b** *Gzmb* (Granzyme B), **c** *Ifng* (IFN-γ), **d** *Prf* (Perforin), **e** *Tbx21* (T-bet), **f** *Cd4* (CD4), **g** *Pdcd1* (PD-1), **h** *Cd274* (PD-L1), **i** *Gata3* (GATA-3). Results pooled from three separate experiments, *n* = 2–12, statistics between indicated groups by 1-way ANOVA followed by LSD post-test: *p < 0.05, **p < 0.01, ***p < 0.001

The flow cytometry data suggested an expansion of R9F-specific CD8α^+^ T cells in response to treatment with DPX-R9F, mCPA and anti-PD-1. We used RT-qPCR to determine if there was a correlating increase in cytotoxicity genes within the TME (Fig. 5). We looked for expression of the following genes associated with cytotoxicity: *Cd8a* (CD8α, Fig. 5a), *Gzmb* (Granzyme B, Fig. 5b), *Ifng* (IFN-γ, Fig. 5c), and *Prf* (Perforin, Fig. 5d). We also assessed the level of the Th1 transcription factor *Tbx21* (T-bet, Fig. 5e) and *Cd4* (CD4, Fig. 5f). None of these genes were increased by anti-PD-1 treatment over untreated or isotype control treated mice. However, they were all increased by DPX/mCPA compared to anti-PD-1 alone. Expression of *Gzmb* was significantly higher in the DPX/mCPA/anti-PD-1 group compared to that in the DPX/mCPA group, and in general the expression of each gene tended to be highest in the group treated with DPX/mCPA/anti-PD-1 combination, which is consistent with the flow cytometry analysis of TILs in the TME.

The most striking increase in mRNA observed was for *Pdcd1* (PD-1, Fig. 5g). For this gene, the level of mRNA was significantly increased by 27.7 times that of the untreated control by DPX/mCPA treatment, and then further increased to 77.7 times that of the untreated control by DPX/mCPA/anti-PD-1 combination treatment. Although expression of *Cd274* (PD-L1, Fig. 5h) was increased by DPX/mCPA treatment relative to that of anti-PD-1 only, it was not further increased by DPX/ mCPA/anti-PD-1.

Finally, we assessed the expression of the Th2 transcription factor *Gata3* (GATA-3, Fig. 5i). Although there were some variations in expression between the different treatment groups, the magnitude of these fluctuations was low (maximum 5-fold).

We noted that for five of the nine genes analysed (*CD8a*, *Gzmb*, *Ifng*, *Prf*, *Tbx21*, and *CD4*) the DPX/ mCPA/isotype control group seemed to have reduced expression compared to DPX/mCPA. Although not significant, this could be an indication that the isotype control (rat anti-mouse) may be having some antigen-non-specific effect on the immune response.

### Anti-PD-1 facilitated the clonal expansion of T cells induced by DPX vaccination and mCPA within the TME

**Fig. 6:**
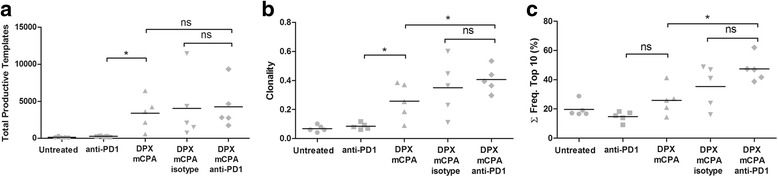
Anti-PD-1 treatment increases the clonality of TILs induced by DPX vaccination and mCPA. Mice were implanted with C3 tumors and treated with 1 week of mCPA commencing 14 days after implantation. Mice were vaccinated on study day 21 and treated with anti-PD-1 or isotype control on study day 26. All mice were terminated on study day 31. Total gDNA was isolated from tumors for TCRβ sequencing. **a** Total productive TCRβ molecules in each sample, **b** Clonality, **c** Cumulative frequency (Σ Freq.) of top 10 most frequent clones. Results from a single experiment, *n* = 5, statistics between indicated groups by 1-way ANOVA followed by LSD post-test: *p < 0.05

Tumor samples taken from clinical trial subjects treated with anti-PD-1 suggested that anti-PD-1 can increase clonality of the TIL populations [19]. Our results thus far have indicated that in combination with DPX and mCPA treatment, anti-PD-1 leads to high levels of antigen-specific CD8α^+^ T cells and increased CTL gene expression within the TME. To determine what effect anti-PD-1 treatment may have on clonal expansion within the TME in the context of an active and targeted immune response, we applied next generation sequencing to analyze intratumoral TCRβ populations using total tumor genomic DNA. These results are shown in Fig. 6.

The total number of TCRβ productive template molecules detected in each sample is a quantitative measurement of the total number of T cells in each sample. As shown in Fig. 6a, treatment with DPX/mCPA increased the number of TCRβ productive template molecules compared to those with anti-PD-1 treatment, and this was not further increased by treatment with DPX/ mCPA/anti-PD-1. Although these TCRβ template molecules represent both CD8^+^ and CD4^+^ T cells, they are consistent with the flow cytometry analysis of the CD8α^+^ TIL population (Fig. 4b) and the mRNA levels of CD8α (Fig. 5a) and CD4 (Fig. 5f). Clonality is a composite measure of both proliferation (frequency) and diversity (uniqueness) of the TCRβ repertoire, and is shown in Fig. 6b. The clonality of the untreated (average 0.069) and anti-PD-1 treatment groups (average 0.085) were low, indicating a highly diverse population of T cells with relatively low clonal proliferation. The clonality of the DPX/mCPA treated group was significantly higher (average 0.258), and the clonality of the DPX/mCPA/anti-PD-1 treated group was the highest (average 0.407). The cumulative frequency of the top ten most expanded clones in the group treated with DPX/mCPA/anti-PD-1 (Fig. 6c) was also the highest, indicating that the top ten most expanded clones in the TME accounted for 46.5 % of all the TILs, whereas this population accounted for 25.5 % in the DPX/mCPA treated group and 14.7 % in the anti-PD-1 treated group. Although the difference between DPX/mCPA and DPX/ mCPA/anti-PD-1 are significant, they are not significant between DPX/mCPA/isotype and DPX/mCPA/ anti-PD-1. We have already demonstrated that the isotype antibody does not increase the antigen-specific response induced by DPX/mCPA (Fig. 4c), and the RT-qPCR data for the DPX/mCPA/isotype control group indicated that the isotype antibody may be having some effect (Fig. 5). Therefore, we think that since the clonality data is based on a single experiment with *n* = 5 the DPX/mCPA is a more relevant control.

**Fig. 7:**
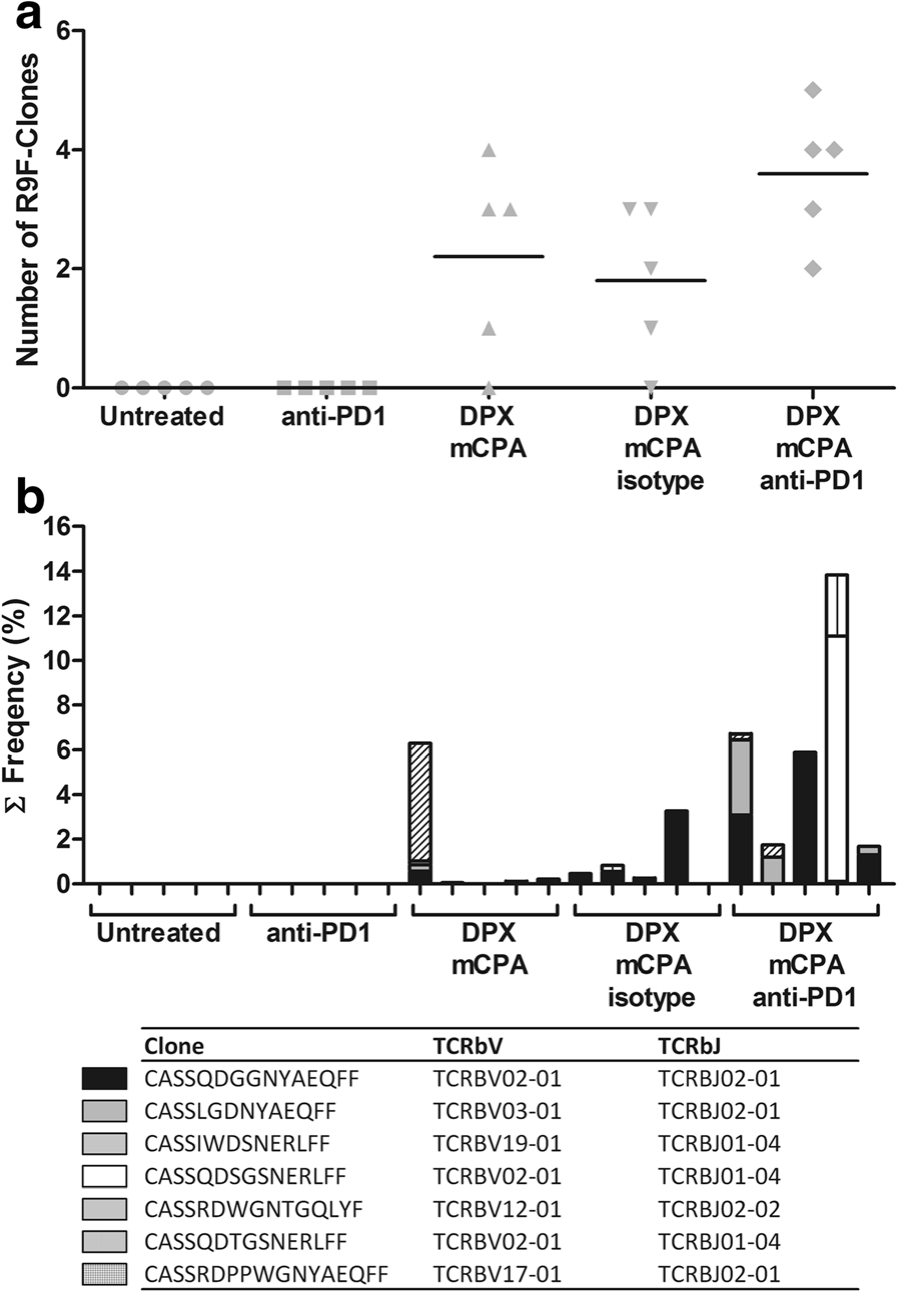
Anti-PD-1 increases the diversity and frequency of antigen-specific clones induced by DPX vaccination and mCPA. A R9F-clone library was created by purifying the R9F-specific CD8α^ +^ T cells clones from the spleens of vaccinated, C3 tumor bearing mice (*n* = 3) using FACS and performing TCRβ sequencing. From the three mice, 26 different clones were identified at a frequency >1 % and considered R9F-specific. These clones were screened for in tumor TCRβ data generated in Fig. 6. **a** Number of different R9F-specific clones from the library identified in each treatment group **b** Frequency of R9F-specific clones

It is not possible to identify the antigen-specificity of the clones by sequence alone. To create a library of possible R9F-specific clones, we vaccinated three C3 tumor bearing mice with DPX-R9F and purified the R9F-specific CD8α^+^ T cells from the spleens 8 days later using FACS; purity of the R9F-specific population was on average 85 %. TCRβ sequencing of these cells revealed 26 unique clones across all three samples with a frequency greater than 1 %, we considered this the threshold for clones that were likely R9F-specific (Additional file 1: Table S2). We screened the TIL populations from the previous experiment using the R9F-clone library. No R9F-specific clones from the library were identified in the untreated control or anti-PD-1 treated groups (Fig. 7a). Mice treated with DPX/mCPA and DPX/mCPA/isotype control had on average 2.2 and 2.0, respectively, unique R9F-specific clones. The highest number of unique clones was detected in the DPX/mCPA/anti-PD-1 combination treated group, which had on average 3.6 R9F-clones in the TME. The cumulative frequency of these R9F-specific clones in the DPX/mCPA and DPX/mCPA/isotype controls groups ranged from 0.000 to 6.285 %, whereas in the DPX/mCPA/anti-PD-1 treated group they ranged from 1.690 to 13.852 % (Fig. 7b).

## Discussion

In summary, we have demonstrated that the use of anti-PD-1 in combination with a potent vaccine-based immunotherapy provides effective control of advanced C3 tumors. Delving into the mechanism, we found that anti-PD-1 treatment enhanced clonal expansion of T cells induced by DPX vaccination and mCPA treatment within the TME. Our results indicate that anti-PD-1 treatment enhances the expansion of antigen-specific CD8α^+^ T cells, and therefore provides a rationale for the combination of PD-1 blockade with active, targeted immune therapy.

Blockade of the PD-1:PD-L1 signaling axis using monoclonal antibodies to either PD-1 or PD-L1 has shown remarkable efficacy in several clinical trials [17, 27]. However, not all patients benefit from treatment, and it is difficult to get consistent responses in different animal tumor models [28]. Although positive PD-L1 expression in the TME is highly correlated with responses to PD-1 blockade [29, 30], approximately 15 % of PD-L1 negative patients also respond to treatment [31]. The difficulty in relying on PD-L1 expression is that there can be temporal and spatial expression variations within a tumor [30], as well as inconsistencies in expression profiles depending on the antibody used for detection. Cancers with high mutational burden and increased TILs have had the most benefit from treatment with PD-1 blockade [17, 21], but many cancer types are not associated with high mutational loads and may not experience the same improvement after anti-PD-1 treatment [32]. The C3 model used in this study has low expression of PD-L1 and TIL infiltration in the absence of treatment; therefore it serves as a useful model for anti-PD-1 treatment of tumors with a negative prognostic profile. Indeed, the C3 tumor was non-responsive to anti-PD-1 treatment. On its own, anti-PD-1 had a minimal effect on increasing infiltrates of CD45^+^ or CD8α^+^ TILs in the TME. Similarly, it did not increase R9F-specific T cells and at the mRNA expression level there was also no increase in four cytotoxic specific genes. A major finding of these experiments was that even in this anti-PD-1 refractory tumor, anti-PD-1 was able to further increase tumor infiltration with antigen-specific R9F cells and T cell clonality expansion in the TME initiated by DPX-R9F and mCPA. Similar findings were also reported by Karyampudi and colleagues using the anti-PD-1 refractory TUBO tumor model, treating with a peptide vaccine and anti-PD-1 [23]. They postulated that anti-PD-1 may encourage epitope spreading, and our results indicate that this may indeed be a factor.

As in other models, in this study we showed that anti-PD-1 in combination with DPX vaccination and mCPA provided enhanced protection against tumor growth [24, 33]. We observed similar results in B16 melanoma and MOSE ovarian tumor models (Additional file 1: Figure S2), however these results do not conclusively demonstrate a significant improvement. This may be in part attributed to the aggressive nature of these tumors. Certainly slower growing tumors may be more likely to respond to treatment, as there is more time for the tumor to interact with the immune system, and also for the immune therapy to stimulate the immune system sufficiently. This is an important consideration for clinical trial design. The translational relevancy of the mouse anti-PD-1 reagents may also contribute to reduced efficacy. The commonly used anti-PD-1 clones for mouse research, RMP1-14 (used in this study) and J43 are rat and hamster antibodies, respectively. However, human anti-PD-1 reagents are human. Repeated injections of foreign antibodies may induce anti-rat antibodies by the murine host, thereby reducing efficacy in long term experiments. All the short term experiments performed in this study used only a single injection of anti-PD-1 so are less likely to be impacted by this effect. However, the inconsistent responses observed in the DPX/ mCPA/isotype control group may be another indication that some non-specific effects are occurring.

Our efforts to deduce if this therapeutic benefit could be attributed to more potent immune responses to the vaccine prompted us to examine the antigen-specific IFN-γ responses by ELISpot in the spleen, vaccine draining lymph node and tumor draining lymph node. We did see an enhancement of immune responses in the spleen, however the responses in the vaccine draining and tumor draining lymph nodes did not show any significant enhancement to vaccine responses with anti-PD-1 treatment. Others have also reported an enhancement of immune responses in the spleen of vaccinated mice also treated with anti-PD-1, but these studies have not also looked at immune responses in the lymph nodes. Recent studies have reported that immune responses assessed in patient PBMCs may not be the best representation of immune responses within the TME, which is arguably the most important site for immune evaluation [20, 34]. Our data also suggest that immune responses can vary within different mouse tissues in response to treatment, and thus may not be useful for correlating to immune responses within the TME. Therefore, we concentrated on evaluating immune responses in the TME using flow cytometry, RT-qPCR and TCRβ sequencing.

Treatment with DPX/mCPA increased TILs compared to anti-PD-1 alone as measured by flow cytometry, and total CD8α^+^ T cells, as measured by RT-qPCR. Combining these therapies resulted in significantly high levels of antigen-specific CD8α^+^ T cells in the TME, yet the accumulation of TILs and CD8a^+^ TILs was not significantly increased. These results indicate that the DPX/mCPA/anti-PD-1 treatment was most efficient in expanding antigen-specific populations of CD8α^+^ T cells within the TME. In the DPX/mCPA/anti-PD-1 treated group, we also detected the highest levels of PD-1 mRNA, which is consistent with a previous report that documented increased PD-1 expression with anti-PD-1 treatment and that likely coincided with retention of T cell functionality and proliferative capabilities within the TME [35].

To our knowledge, this is the first study to use a pre-clinical model to investigate the effects of vaccination, mCPA and anti-PD-1 combination therapy on the clonality of T cells within the TME. We have found that the C3 model is useful for studying immune modulation of antigen-specific immune responses and their effect on tumor growth. Our previous work with this tumor model provided insight into immune modulatory effects between DPX vaccination and mCPA, which were applied in designing scheduling and immune monitoring in our clinical trial. In this study, the tumor model has indicated that next generation sequencing to evaluate T cell clonality may be a useful tool to evaluate the combination of vaccination with PD-1 blockade. Increased clonality has recently been indicated as a predictive biomarker of response to anti-PD-1 monotherapy in a clinical trial [36] and PD-1^+^CD8^+^ TIL population has been shown to consist of clonally expanded, tumor-reactive T cells [20]. Our results indicate that combining anti-PD-1 with vaccination increases the clonal expansion of select clones, resulting in a less diverse T cell population represented by a few highly expanded clones. Therefore, clonality may be a useful biomarker to assess immune responses in clinical trials after anti-PD-1 and vaccination therapy.

We attempted to identify R9F-specific clones by creating a clone library using R9F-specific CD8α^+^ T cells purified from three DPX-R9F vaccinated mice. From these three mice, a total of 26 putative R9F-specific clones were identified, but only three of these clones were detected in all three mice and at different frequencies. Due to the poor correlation of highly frequent clones between the three populations, we must conclude that the library generated was not an extensive representation of all R9F-specific clones. The variation observed in this small population suggests that the expansion of antigen-specific clones is unique to individual mice, and a larger population would be necessary in order to obtain a complete library. However, this degree of variation that we observed among syngeneic mice vaccinated with an immunodominant epitope also suggests that identification of antigen-specific clones in a genetically diverse human population would be difficult. Therefore, the clonality measurement of whole tumor gDNA may be a more comparable statistic to use when evaluating the effect of immune therapy on T cell expansion. Clonal analysis is an important tool that can be used to understand complex immune responses using limited sample material. Future directions for this work could involve more extensive investigation into the antigen-specificity of specific clones in order to identify the higher affinity clones and then track their development following immune therapy. Combinations with the vaccine and other clinical antibody candidates, such as anti-OX40 and anti-GITR, may be interesting comparators to anti-PD-1 as they promote T cell activation by directly providing stimulation, rather than blocking suppression.

## Conclusion

Our results indicate that anti-PD-1 therapy compliments the strong antigen-specific, cytotoxic immune responses induced by DPX vaccination and mCPA. This combination may improve responses in subjects that are non-responsive to anti-PD-1 treatment alone, or have low pre-existing intratumoral PD-L1 expression. PD-1 blockade promotes effective immune responses within the TME by enhancing the clonal expansion of antigen-specific CD8α^+^ T cells.

## Additional file

Additional file 1: Table S1. Primer sequences. **Table S2**. R9F-clone library. C3 tumor bearing mice (C57BL/6, *n* = 3) were treated with mCPA for 1 week and then vaccinated with DPX-R9F. Eight days later, mice were euthanized and spleens collected. R9F-specific CD8α + T cells were purified from spleens using FACS and TCRβ sequencing performed by Adaptive Biotechnologies. From the three mice, 26 different clones were identified at a frequency >1 % and considered R9F-specific. The table lists the amino acid sequence of each clone, the TCRbV and TCRbJ gene associated with each clone, and the frequency of each clone among the three samples. Highlighted in grey are the clones found in all samples. Highlighted in red are the most frequent clones of each sample. **Figure S1**. Upregulation of surface antigens on C3 tumor cells in vitro after culture with IFN-γ (50 U/mL, 48 hours). Surface expression determined by flow cytometry. Results representative of 3 separate experiments. Black: isotype control, blue: unstimulated, red: IFN-γ stimulated. **Figure S2**. (A, B) C57BL6 mice (*n* = 10) were implanted with B16-F10 tumors and treated with mCPA starting on day 3 for one week one, 1 week off. Mice were vaccinated with DPX containing the TRP2180-188 peptide (DPX-S9L) on days 3 and 17. Anti-PD-1 or isotype control administered on days 3, 6, 9, 17, 20, 23. (C) HLA-A2 transgenic mice (*n* = 5) were implanted subcutaneously with syngeneic ovarian tumors and treated using the same schedule as the B16-F10, except that mice were vaccinated with DPX-Survivac. **Figure S3**. Immunohistochemical staining of tumor sections for CD45 and CD8α expression. Samples were cryoprotected and snap frozen. Sections were fixed and blocked, then treated with anti-CD8α or anti-CD45 followed by biotinylated anti-rat IgG. Staining was visualized using Vectastain ABC kit (Vector Laboratories) and counterstained with Gill’s Hematoxylin. Microscopy was performed at a 10X magnification. (PPTX 478 kb)

## Abbreviations

DPX: DepoVax
mCPA: Metronomic cyclophosphamide
PD-1: Programmed death 1 receptor
RT-qPCR: Reverse transcription quantitative polymerase chain reaction
TIL: Tumor infiltrating lymphocytes
TME: Tumor microenvironment
