# Supplementary material for: Anti-PD-1 increases the clonality and activity of tumor infiltrating antigen specific T cells induced by a potent immune therapy consisting of vaccine and metronomic cyclophosphamide
Source: J Immunother Cancer. 2016 Oct 18;4:68. doi: 10.1186/s40425-016-0169-2 (PMC5067905; doi:10.1186/s40425-016-0169-2)
Supplement: Additional file 1: Table S1. — Primer sequences. Table S2. R9F-clone library. C3 tumor bearing mice (C57BL/6, n = 3) were treated with mCPA for 1 week and then vaccinated with DPX-R9F. Eight days later, mice were euthanized and spleens collected. R9F-specific CD8α + T cells were purified from spleens using FACS and TCRβ sequencing performed by Adaptive Biotechnologies. From the three mice, 26 different clones were identified at a frequency >1 % and considered R9F-specific. The table lists the amino acid sequence of each clone, the TCRbV and TCRbJ gene associated with each clone, and the frequency of each clone among the three samples. Highlighted in grey are the clones found in all samples. Highlighted in red are the most frequent clones of each sample. Figure S1. Upregulation of surface antigens on C3 tumor cells in vitro after culture with IFN-γ (50 U/mL, 48 hours). Surface expression determined by flow cytometry. Results representative of 3 separate experiments. Black: isotype control, blue: unstimulated, red: IFN-γ stimulated. Figure S2. (A, B) C57BL6 mice (n = 10) were implanted with B16-F10 tumors and treated with mCPA starting on day 3 for one week one, 1 week off. Mice were vaccinated with DPX containing the TRP2180-188 peptide (DPX-S9L) on days 3 and 17. Anti-PD-1 or isotype control administered on days 3, 6, 9, 17, 20, 23. (C) HLA-A2 transgenic mice (n = 5) were implanted subcutaneously with syngeneic ovarian tumors and treated using the same schedule as the B16-F10, except that mice were vaccinated with DPX-Survivac. Figure S3. Immunohistochemical staining of tumor sections for CD45 and CD8α expression. Samples were cryoprotected and snap frozen. Sections were fixed and blocked, then treated with anti-CD8α or anti-CD45 followed by biotinylated anti-rat IgG. Staining was visualized using Vectastain ABC kit (Vector Laboratories) and counterstained with Gill’s Hematoxylin. Microscopy was performed at a 10X magnification. (PPTX 478 kb) [file 40425_2016_169_MOESM1_ESM.pptx]

## Slide 1
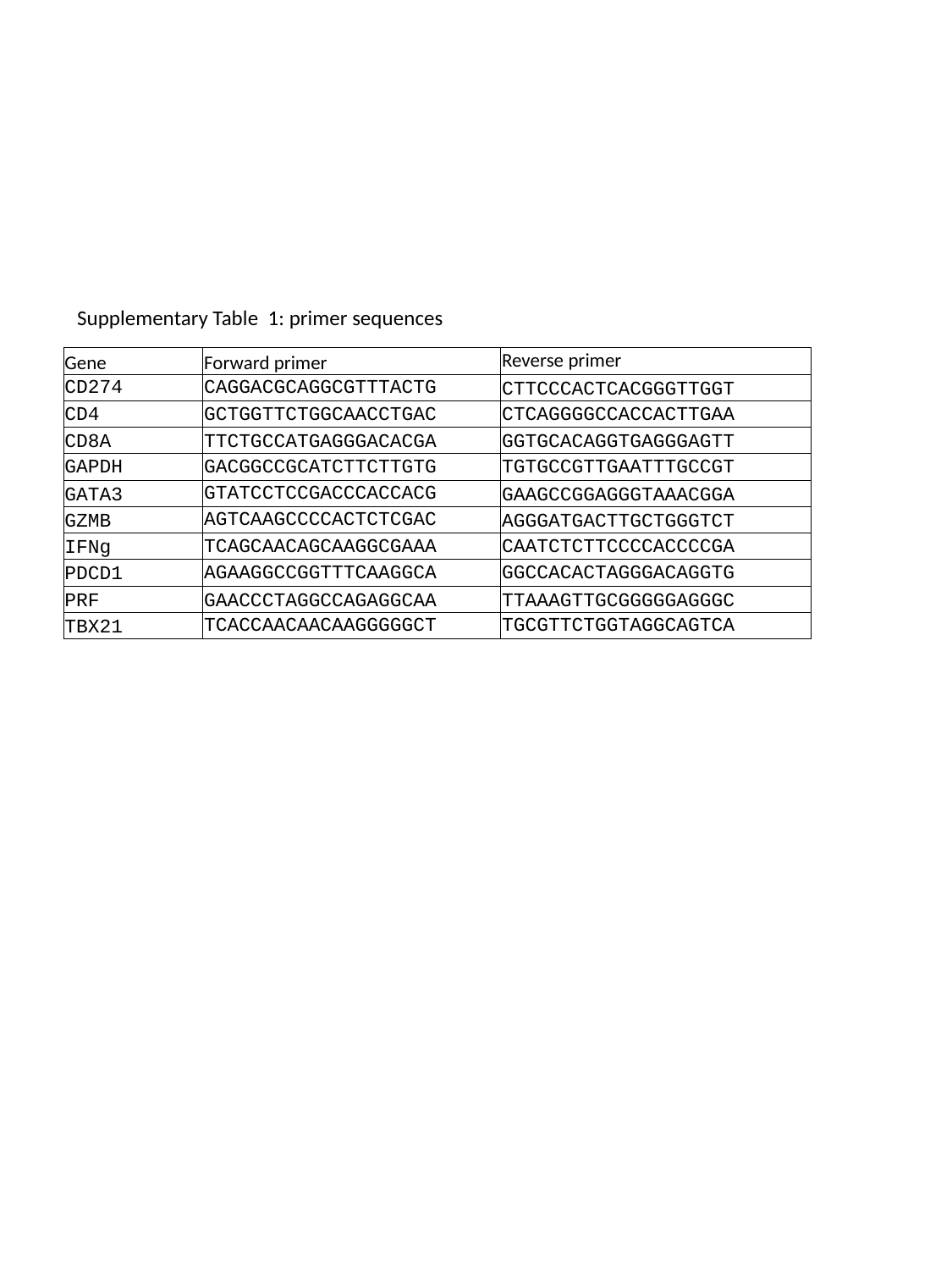

Supplementary Table 1: primer sequences
| Gene | Forward primer | Reverse primer |
| --- | --- | --- |
| CD274 | CAGGACGCAGGCGTTTACTG | CTTCCCACTCACGGGTTGGT |
| CD4 | GCTGGTTCTGGCAACCTGAC | CTCAGGGGCCACCACTTGAA |
| CD8A | TTCTGCCATGAGGGACACGA | GGTGCACAGGTGAGGGAGTT |
| GAPDH | GACGGCCGCATCTTCTTGTG | TGTGCCGTTGAATTTGCCGT |
| GATA3 | GTATCCTCCGACCCACCACG | GAAGCCGGAGGGTAAACGGA |
| GZMB | AGTCAAGCCCCACTCTCGAC | AGGGATGACTTGCTGGGTCT |
| IFNg | TCAGCAACAGCAAGGCGAAA | CAATCTCTTCCCCACCCCGA |
| PDCD1 | AGAAGGCCGGTTTCAAGGCA | GGCCACACTAGGGACAGGTG |
| PRF | GAACCCTAGGCCAGAGGCAA | TTAAAGTTGCGGGGGAGGGC |
| TBX21 | TCACCAACAACAAGGGGGCT | TGCGTTCTGGTAGGCAGTCA |

## Slide 2
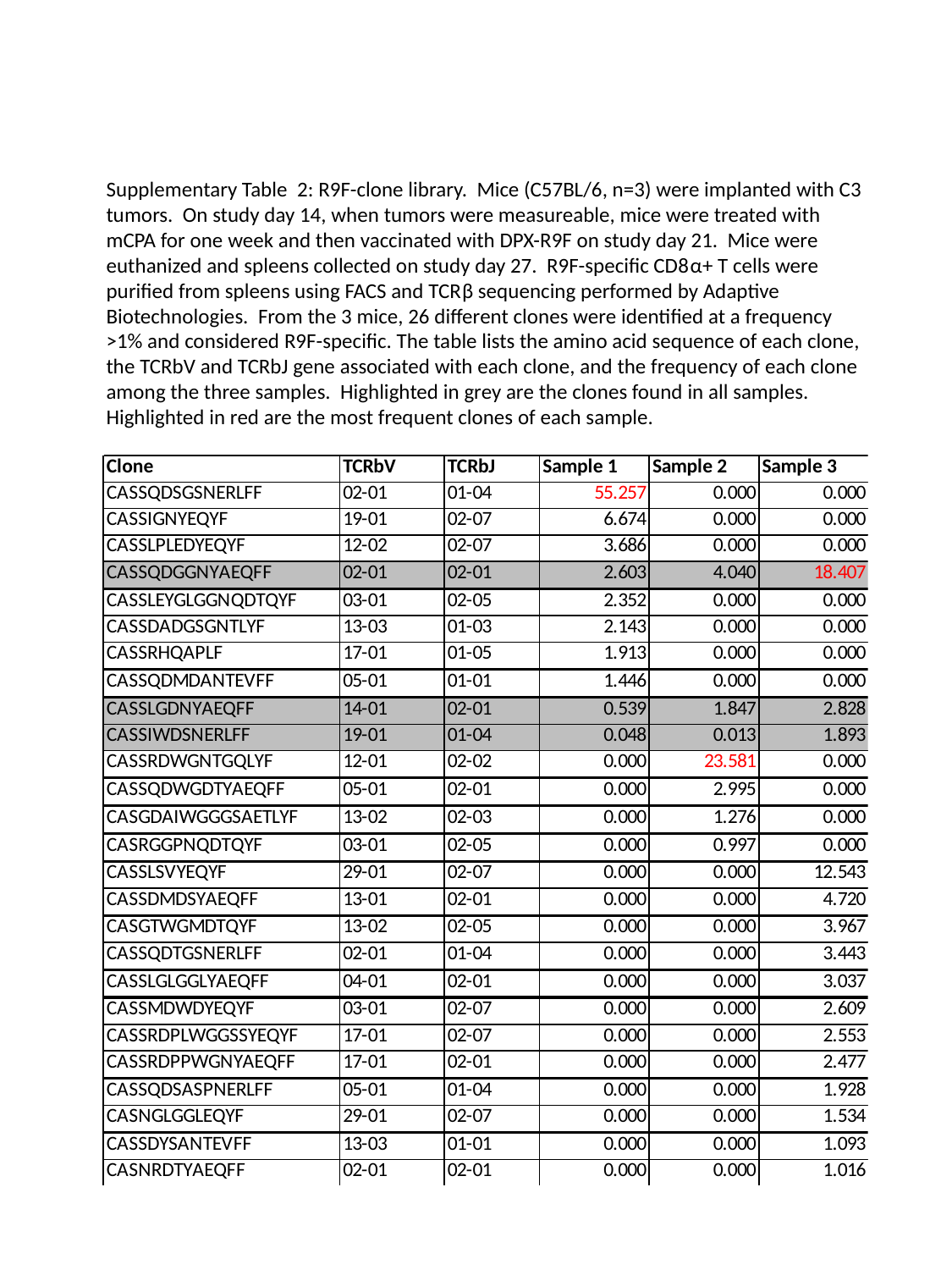

Supplementary Table 2: R9F-clone library. Mice (C57BL/6, n=3) were implanted with C3 tumors. On study day 14, when tumors were measureable, mice were treated with mCPA for one week and then vaccinated with DPX-R9F on study day 21. Mice were euthanized and spleens collected on study day 27. R9F-specific CD8α+ T cells were purified from spleens using FACS and TCRβ sequencing performed by Adaptive Biotechnologies. From the 3 mice, 26 different clones were identified at a frequency >1% and considered R9F-specific. The table lists the amino acid sequence of each clone, the TCRbV and TCRbJ gene associated with each clone, and the frequency of each clone among the three samples. Highlighted in grey are the clones found in all samples. Highlighted in red are the most frequent clones of each sample.

## Slide 3
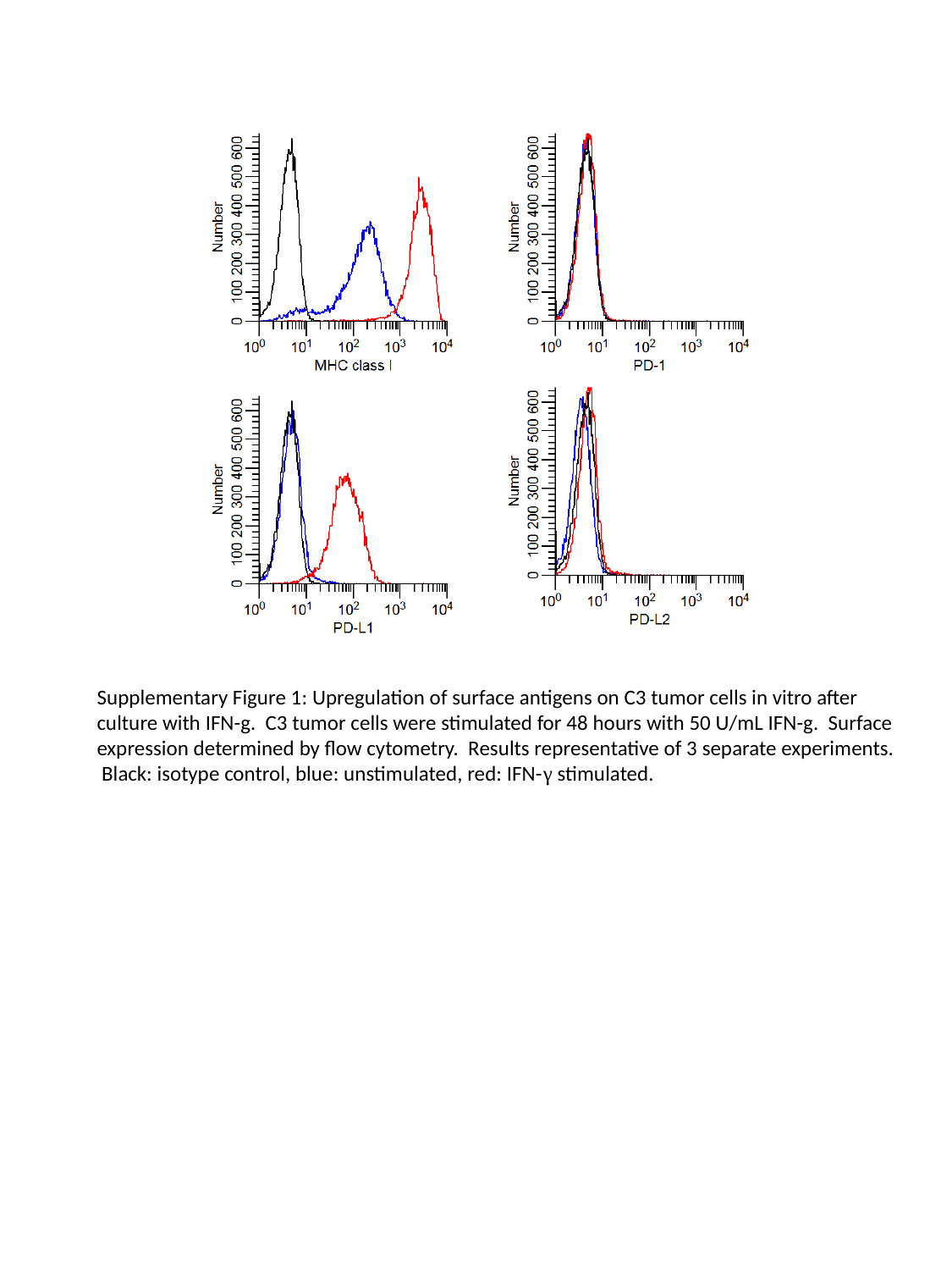

Supplementary Figure 1: Upregulation of surface antigens on C3 tumor cells in vitro after culture with IFN-g. C3 tumor cells were stimulated for 48 hours with 50 U/mL IFN-g. Surface expression determined by flow cytometry. Results representative of 3 separate experiments. Black: isotype control, blue: unstimulated, red: IFN-γ stimulated.

## Slide 4
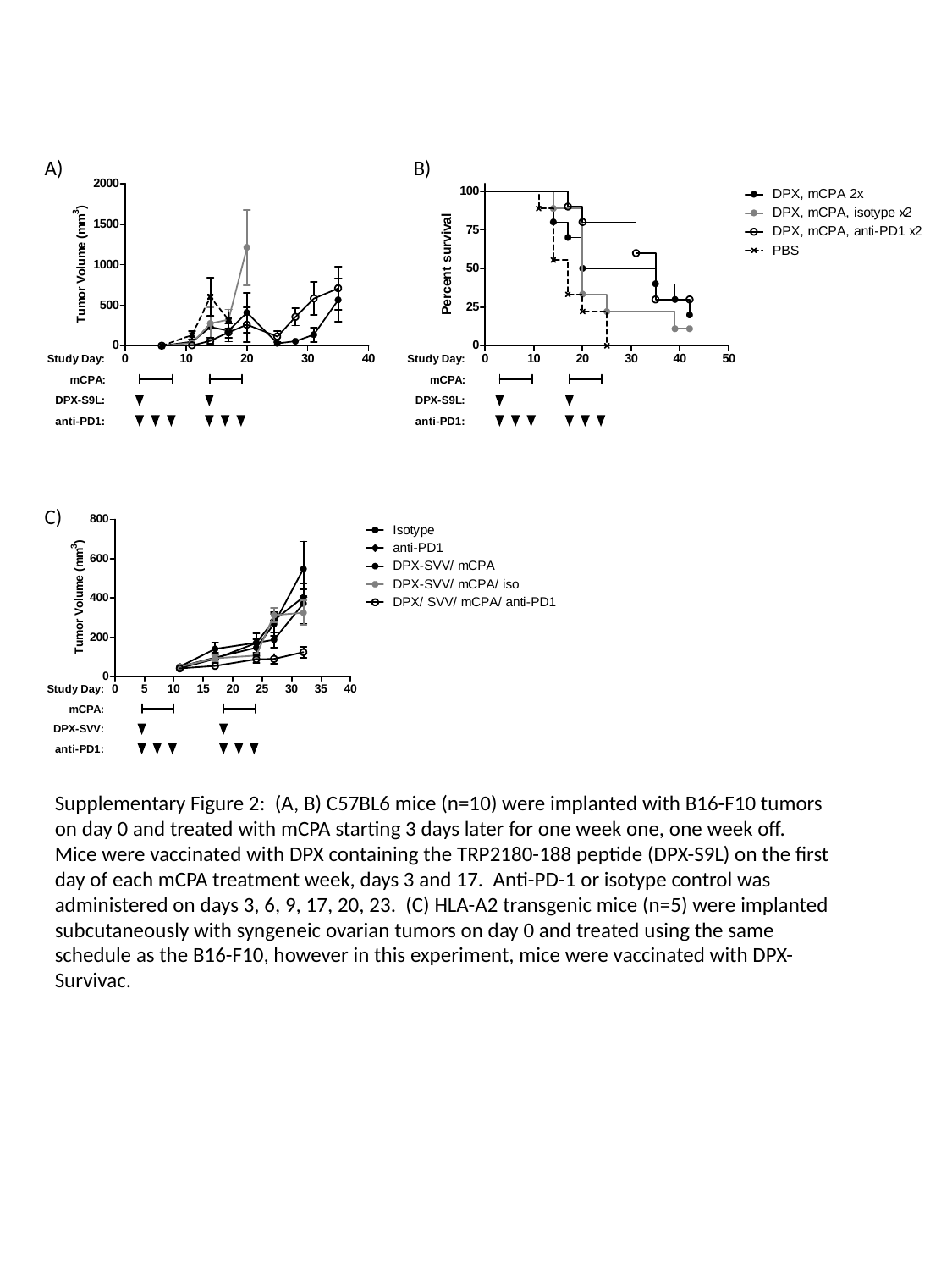

A)
B)
C)
Supplementary Figure 2: (A, B) C57BL6 mice (n=10) were implanted with B16-F10 tumors on day 0 and treated with mCPA starting 3 days later for one week one, one week off. Mice were vaccinated with DPX containing the TRP2180-188 peptide (DPX-S9L) on the first day of each mCPA treatment week, days 3 and 17. Anti-PD-1 or isotype control was administered on days 3, 6, 9, 17, 20, 23. (C) HLA-A2 transgenic mice (n=5) were implanted subcutaneously with syngeneic ovarian tumors on day 0 and treated using the same schedule as the B16-F10, however in this experiment, mice were vaccinated with DPX-Survivac.

## Slide 5
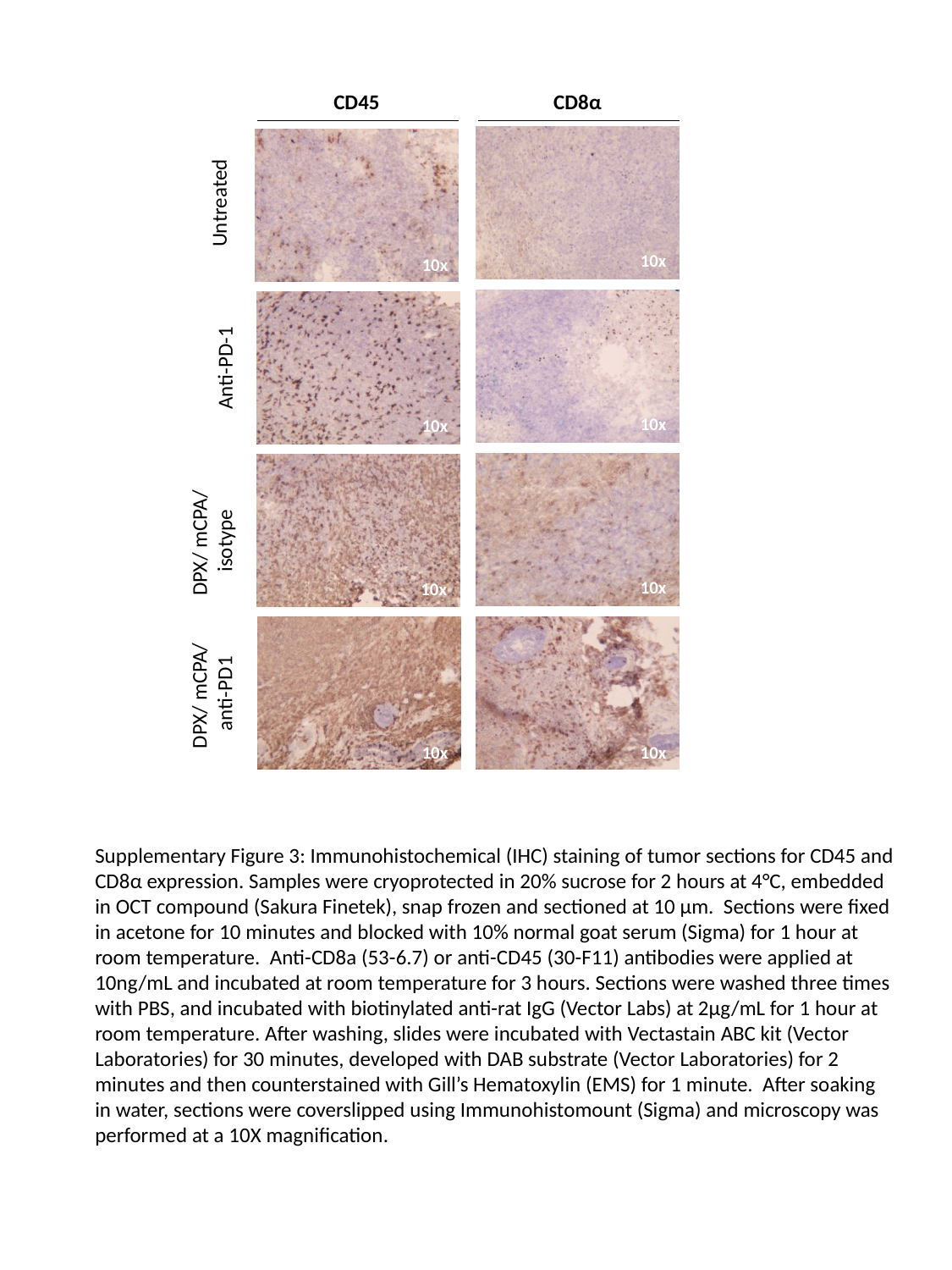

CD45
CD8α
Untreated
10x
10x
Anti-PD-1
10x
10x
DPX/ mCPA/
isotype
10x
10x
DPX/ mCPA/
anti-PD1
10x
10x
Supplementary Figure 3: Immunohistochemical (IHC) staining of tumor sections for CD45 and CD8α expression. Samples were cryoprotected in 20% sucrose for 2 hours at 4°C, embedded in OCT compound (Sakura Finetek), snap frozen and sectioned at 10 µm. Sections were fixed in acetone for 10 minutes and blocked with 10% normal goat serum (Sigma) for 1 hour at room temperature. Anti-CD8a (53-6.7) or anti-CD45 (30-F11) antibodies were applied at 10ng/mL and incubated at room temperature for 3 hours. Sections were washed three times with PBS, and incubated with biotinylated anti-rat IgG (Vector Labs) at 2µg/mL for 1 hour at room temperature. After washing, slides were incubated with Vectastain ABC kit (Vector Laboratories) for 30 minutes, developed with DAB substrate (Vector Laboratories) for 2 minutes and then counterstained with Gill’s Hematoxylin (EMS) for 1 minute. After soaking in water, sections were coverslipped using Immunohistomount (Sigma) and microscopy was performed at a 10X magnification.
